# Draft Genome Sequence of *Saccharomyces cerevisiae* Strain NCIM3186 Used in the Production of Bioethanol from Sweet Sorghum

**DOI:** 10.1128/genomeA.00813-15

**Published:** 2015-07-30

**Authors:** Burragoni Sravanthi Goud, Kandasamy Ulaganathan

**Affiliations:** Centre for Plant Molecular Biology, Osmania University, Hyderabad, Telangana, India

## Abstract

Here, we report the draft genome sequence of *Saccharomyces cerevisiae* strain NCIM3186 used in bioethanol production from sweet sorghum. The size of the genome is approximately 11.9 Mb and contains 5,347 protein-coding genes.

## GENOME ANNOUNCEMENT

*Saccharomyces cerevisiae* is the most preferred host organism for lignocellulosic bioethanol production due to its ability in fermentative production of high ethanol, inhibitor tolerance, and suitability for heterologous expression of genes (1–3). Understanding the genomic variations that facilitate high ethanol production by *S. cerevisiae* is necessary for engineering strains for lignocellulosic bioethanol production. Many strains used in bioethanol production have been sequenced, and a number of variations have been identified (4–8). In our effort to select a suitable strain for lignocellulosic bioethanol production, we have sequenced strains differing in their ability to produce bioethanol from plant biomass and reported the genome sequence of a moderate bioethanol-producing strain, NCIM3107 (9). Here, we report the draft genome sequence of *S. cerevisiae* strain NCIM3186 used in bioethanol production from sweet sorghum (10).

*S. cerevisiae* strain NCIM3186 was obtained from the Microbial Type Culture Collection, Chandigarh, India. Genome sequencing was done using the Illumina MiSeq sequencing platform (Illumina, San Diego, CA). The sequencing generated paired-end read files R1 and R2 consisting of a total of 15,216,196 reads. Quality assessment was performed using FastQC (http://www.bioinformatics.babraham.ac.uk/projects/fastqc/) and adapter trimming by the FASTX-Toolkit (http://hannonlab.cshl.edu/fastx_toolkit/). The genome assembly was performed by using both *de novo* and reference-based genome assemblers. The *S. cerevisiae* S288C R64-1.1 reference genome was used to perform a reference-based assembly of raw reads by using Bowtie 2 (11), which generated a SAM alignment file as output. Using this SAM file BAM file, sorted BAM and indexed BAM files were made using SAMtools (12). The overall coverage was estimated to be 87.7×. *De novo* assembly of reads was performed using the Velvet assembler (13). Using SAMtools, a consensus sequence of size 11.9 Mb was created. Variant calling was performed using *S. cerevisiae* S288C R64-1.1 reference genome and ≥20-read depth and ≥30 mapping quality score as cutoffs. Further annotation of variants was done using SnpEFF 3.4 (14). Gene prediction was done by using Augustus (15). A total of 5,347 protein-coding genes were found, which showed homology to *S. cerevisiae* genes in the *Saccharomyces* Genome Database (SGD) (16).

### Nucleotide sequence accession numbers.

The nucleotide sequences of the NCIM3186 strain of the *S. cerevisiae* genome have been deposited in GenBank under the accession numbers CP011810 to CP011825 and the mitochondrial genome under the accession no. CP011826.
